# Association of Early Postoperative Biochemical Markers with a Composite Adverse Perioperative Outcome After Bariatric Surgery

**DOI:** 10.3390/diagnostics16152332

**Published:** 2026-07-25

**Authors:** Bahar Uslu Bayhan, Tugçe Gazioglu Kisi, Gulay Akinci, Muhammed Gokhan Abay, Bulent Sultanoglu

**Affiliations:** 1 Department of Anesthesiology and Reanimation, Gaziantep City Hospital, Gaziantep 27010, Türkiye; tugce.gazioglu@inonu.edu.tr (T.G.K.); drgulayakinci27@gmail.com (G.A.); abay.25@gmail.com (M.G.A.); 2 Department of General Surgery, Gaziantep City Hospital, Gaziantep 27010, Türkiye; drbulentsultan@hotmail.com

**Keywords:** bariatric surgery, postoperative complications, biomarkers, lactate-to-albumin ratio, aspartate aminotransferase, postoperative outcomes

## Abstract

**Background/Objectives:** Bariatric surgery is the most effective treatment for sustained weight loss; however, early postoperative complications remain a significant concern. Routine biochemical markers obtained in the immediate postoperative period may enable early risk stratification, yet their prognostic value remains incompletely defined. This study aimed to evaluate the association between early postoperative biochemical alterations and composite adverse outcomes after bariatric surgery. **Methods:** In this retrospective observational study, 260 adult patients undergoing elective bariatric surgery were included. Preoperative and postoperative day 1 (POD1) laboratory parameters were retrieved from electronic medical records. The primary endpoint was a composite adverse outcome comprising postoperative intensive care unit admission, infection, anastomotic leak, or 30-day readmission. Univariable and multivariable logistic regression analyses were performed to evaluate independent associations, and receiver operating characteristic (ROC) curve analysis was used to assess discriminative performance. **Results:** Composite adverse outcomes occurred in 51 patients (19.6%). In multivariable analysis, longer operative duration (OR 1.17 per 10 min, 95% CI 1.04–1.31; *p* = 0.009), POD1 AST (OR 1.15 per 10 U/L, 95% CI 1.07–1.24; *p* < 0.001), and POD1 lactate-to-albumin ratio (LAR ×100) (OR 1.20, 95% CI 1.04–1.38; *p* = 0.014) were independently associated with adverse outcomes. POD1 AST demonstrated the highest discriminative ability (AUC 0.733), followed by glucose (AUC 0.688) and ALT (AUC 0.680), whereas LAR showed limited stand-alone performance (AUC 0.594). **Conclusions:** Longer operative duration and higher POD1 AST and LAR levels were independently associated with the composite adverse perioperative outcome. AST showed the highest, although moderate, individual discrimination, whereas LAR demonstrated limited stand-alone performance. Because immediate postoperative ICU transfer preceded POD1 biomarker sampling and dominated the composite outcome, these findings should be interpreted as associations rather than prospective prediction.

## 1. Introduction

Obesity has become one of the most important global health challenges, with the World Health Organization estimating that approximately 16% of adults worldwide were living with obesity in 2022, representing more than a twofold increase since 1990. In parallel, the prevalence of metabolic syndrome has risen substantially, affecting an estimated 1.54 billion adults globally by 2023. Owing to its close association with type 2 diabetes, cardiovascular disease, obstructive sleep apnea and metabolic dysfunction-associated steatotic liver disease, obesity is accompanied by a considerable burden of chronic comorbidities. Large population-based studies have shown that more than 60% of individuals with obesity have at least one obesity-related condition. Consequently, these patients are exposed to increased perioperative risk, including higher rates of hemodynamic instability, infectious complications and postoperative organ dysfunction [1,2].

Bariatric surgery remains the most effective long-term treatment for achieving sustained weight loss and remission of obesity-related comorbidities. Nevertheless, surgical trauma and the accompanying physiological stress response induce substantial alterations in inflammatory markers, acute-phase reactants and organ function parameters during the early postoperative period. The ability to utilize these routinely available laboratory biomarkers for early risk stratification may improve patient safety and optimize perioperative resource allocation [3,4,5].

Previous studies have suggested that readily available inflammatory biomarkers may aid in early risk stratification after bariatric surgery. Elevated neutrophil-to-lymphocyte ratio (NLR) has been associated with adverse 30-day outcomes, while both NLR and platelet-to-lymphocyte ratio (PLR) have been linked to postoperative intensive care unit admission and prolonged hospital stay, supporting the role of hematological inflammatory indices in perioperative risk assessment [6,7]. Furthermore, systematic reviews have demonstrated that preoperative hypoalbuminemia and perioperative changes in inflammatory biomarkers may contribute to the prediction of postoperative complications. More recently, the lactate-to-albumin ratio (LAR), which integrates tissue hypoperfusion with systemic inflammatory burden, has emerged as a promising prognostic marker in critically ill and surgical populations [8,9,10]. However, evidence regarding the clinical significance of LAR in bariatric surgery remains limited [11].

Despite growing interest in perioperative biomarkers, current evidence remains heterogeneous with respect to optimal timing, threshold values and the combined use of multiple markers, precluding the establishment of a consistent risk stratification framework. Therefore, the present study aimed to evaluate the association between perioperative biochemical parameters and early clinical outcomes after bariatric surgery. We hypothesized that postoperative alterations in these biomarkers would be independently associated with adverse early clinical outcomes.

## 2. Materials and Methods

### 2.1. Study Design and Patient Population

This retrospective observational study was conducted at the Department of Anesthesiology and Reanimation, Gaziantep City Hospital. The study protocol was approved by the Gaziantep City Hospital Non-Interventional Clinical Research Ethics Committee (Approval No. 479/2026; 18 March 2026) and was conducted in accordance with the principles of the Declaration of Helsinki. Owing to the retrospective design and the use of anonymized patient data, the requirement for informed consent was waived by the ethics committee.

Adult patients who underwent elective bariatric surgery at Gaziantep City Hospital were retrospectively identified from the hospital information system. Patients undergoing sleeve gastrectomy, gastric bypass, or other metabolic/bariatric procedures with complete preoperative and early postoperative clinical and laboratory data were eligible for inclusion.

Patients were excluded if they were younger than 18 years, underwent emergency surgery, had active infection or sepsis at the time of surgery, advanced chronic kidney disease (stage 4–5), advanced liver failure, or underwent a concomitant major surgical procedure other than bariatric surgery. Patients were also excluded when the clinical or laboratory information required to determine the primary outcome or perform the planned analyses remained unavailable after comprehensive review of the electronic medical records. Records containing substantial inconsistencies or unverifiable data were excluded from the analysis.

### 2.2. Data Collection

Demographic and clinical variables were extracted from electronic medical records. Age, sex, height, body weight, body mass index (BMI), American Society of Anesthesiologists (ASA) physical status classification, smoking status, and comorbidities were recorded. Comorbidities included diabetes mellitus, hypertension, obstructive sleep apnea, coronary artery disease, chronic pulmonary disease, non-alcoholic fatty liver disease, and hypothyroidism.

Available perioperative variables included the type of bariatric procedure, surgical approach, and operative duration. Perioperative management was guided by the institutional enhanced recovery after surgery protocol routinely applied at our center. However, detailed information regarding the specific anesthetic agents and cumulative doses, the duration and magnitude of intraoperative hypotension, cumulative vasopressor dosage, net intraoperative fluid balance, estimated blood loss, and urine output was not consistently available in structured electronic records and therefore could not be included in the analysis.

### 2.3. Laboratory Parameters and Derived Indices

Preoperative and postoperative day 1 (POD1) laboratory results obtained as part of routine clinical practice were retrieved from the electronic database. Evaluated parameters included hemoglobin, hematocrit, white blood cell count, neutrophil count, lymphocyte count, platelet count, C-reactive protein (CRP), albumin, creatinine, blood urea nitrogen, aspartate aminotransferase (AST), alanine aminotransferase (ALT), sodium, potassium, glucose, and lactate levels.

The neutrophil-to-lymphocyte ratio (NLR) was calculated by dividing the absolute neutrophil count by the absolute lymphocyte count. The lactate-to-albumin ratio (LAR) was calculated by dividing serum lactate by serum albumin measured at the same time point. NLR and LAR were calculated separately using both preoperative and POD1 values. Since the primary objective of this study was to investigate the prognostic significance of early postoperative biochemical alterations, the analyses focused mainly on POD1 biomarkers. In addition, perioperative changes in hemoglobin, creatinine, and albumin concentrations were calculated as the difference between POD1 and preoperative values (Δ value = POD1 value − preoperative value).

### 2.4. Outcome Definitions

The primary endpoint was a composite adverse clinical outcome, defined as the occurrence of at least one of the following events: postoperative intensive care unit admission, postoperative infection, anastomotic leak, or hospital readmission within 30 days after discharge. The composite endpoint was selected to capture a broad spectrum of clinically relevant early postoperative events reflecting escalation of care or subsequent healthcare utilization, because individual major complications were relatively uncommon in this elective cohort. The components were not weighted, and the occurrence of any one component classified the patient as having experienced the composite outcome. Because these components differ in clinical severity, their frequencies were also reported separately.

At our institution, postoperative ICU admission was not routinely required for all patients undergoing bariatric surgery. Immediate postoperative ICU transfer was performed when either the attending anesthesiologist or surgeon considered higher-level monitoring or treatment necessary. Clinical considerations included severe obstructive sleep apnea or other major preoperative comorbidities, higher ASA physical status, perioperative respiratory compromise, delayed recovery from anesthesia, requirement for postoperative ventilatory or vasoactive support, hemodynamic instability, significant bleeding, and suspected or recognized surgical complications. A recommendation by either specialist was sufficient for ICU admission, as the anesthesiologist and surgeon assessed different aspects of perioperative risk. Because of the retrospective design and variability in clinical documentation, ICU admissions anticipated preoperatively could not be reliably distinguished from unplanned admissions prompted by intraoperative or immediate postoperative events. All ICU transfers occurred immediately after surgery and before POD1 blood sampling.

Secondary endpoints included intensive care unit admission, postoperative infection, anastomotic leak, length of hospital stay, and 30-day readmission.

### 2.5. Statistical Analysis

Statistical analyses were performed using IBM SPSS Statistics version 26.0 (IBM Corp., Armonk, NY, USA). The distribution of continuous variables was assessed using histograms, Q-Q plots, and the Shapiro–Wilk test. Normally distributed variables are presented as mean ± standard deviation, whereas non-normally distributed variables are expressed as median (interquartile range). Categorical variables are presented as numbers and percentages.

Preoperative and postoperative laboratory parameters were compared using paired-samples t-tests or Wilcoxon signed-rank tests, as appropriate. Comparisons between patients with and without composite adverse outcomes were performed using independent-samples t-tests or Mann–Whitney U tests for continuous variables and χ^2^ or Fisher’s exact tests for categorical variables.

After re-evaluation of the electronic medical records, the dataset of the 260 included patients contained complete data for all variables used in the planned analyses. Therefore, no missing-data imputation was performed, and all descriptive, regression, and ROC analyses were based on the complete cohort of 260 patients.

Univariable logistic regression analyses were initially performed to evaluate the unadjusted associations between candidate clinical, procedural, and biochemical variables and the composite adverse outcome. The results of these analyses are provided in Supplementary Table S1. The multivariable analysis was designed as a parsimonious explanatory model rather than a comprehensive prediction model. Because 51 patients experienced the composite outcome, the number of covariates was restricted to four to reduce the risk of overfitting. BMI was retained as a clinically relevant baseline variable, and operative duration was included to represent procedural burden. Among the postoperative biomarkers, AST was selected as the principal marker of hepatocellular and perioperative tissue stress because it demonstrated the strongest univariable association and discriminative performance. LAR was retained as the principal composite perfusion–reserve biomarker evaluated in the study. These four covariates were entered simultaneously into the model. Biologically overlapping or mathematically related variables were not entered together; therefore, ALT was not included alongside AST, and lactate and albumin were not included alongside LAR. Other biomarkers associated with the outcome in univariable analyses were not added because of the limited number of outcome events and the resulting risk of model overfitting. Results are presented as adjusted odds ratios (ORs) with corresponding 95% confidence intervals (CIs).

Receiver operating characteristic (ROC) curve analysis was performed to evaluate the discriminative ability of individual biomarkers, and the area under the curve (AUC) was calculated. The 95% confidence intervals for the AUCs and pairwise comparisons between correlated ROC curves were calculated using the DeLong method. Optimal cut-off values were determined using the Youden index. All statistical tests were two-sided, and a *p* value < 0.05 was considered statistically significant.

## 3. Results

A total of 291 patient records were screened for eligibility. Of these, 31 were excluded: 24 because of incomplete laboratory data and seven because of incomplete clinical records. No patient was excluded because of a concomitant major surgical procedure. The remaining 260 patients were included in the final analysis. The cohort comprised 257 patients undergoing laparoscopic sleeve gastrectomy (98.8%), two undergoing laparoscopic Roux-en-Y gastric bypass (0.8%), and one undergoing laparoscopic one-anastomosis gastric bypass (0.4%). No patient underwent an open or revisional procedure. The mean age was 34.2 ± 10.1 years, and the mean body mass index (BMI) was 46.0 ± 5.0 kg/m^2^. Overall, 51 patients (19.6%) experienced the predefined composite adverse outcome, defined as the occurrence of at least one of the following events: intensive care unit admission, postoperative infection, anastomotic leak, or 30-day hospital readmission. Baseline demographic and clinical characteristics are presented in Table 1.

Significant postoperative alterations were observed across all evaluated laboratory parameters. POD1 was characterized by a marked inflammatory response, reflected by increases in WBC count, neutrophil count, CRP, NLR, lactate, AST, ALT, glucose, and LAR, whereas hemoglobin, platelet count, lymphocyte count, and albumin levels significantly decreased (all *p* < 0.001) (Table 2).

Postoperative ICU admission was the most frequent adverse event, occurring in 41 of the 51 patients with a composite adverse outcome (80.4%), whereas anastomotic leak and postoperative infection were uncommon. Because some patients experienced more than one component, the sum of the individual event counts exceeded the number of patients classified as having the composite outcome. Detailed early postoperative outcomes are summarized in Table 3.

Compared with patients without adverse outcomes, those who experienced composite adverse events had significantly longer operative times and higher POD1 WBC, creatinine, AST, ALT, glucose, lactate, and LAR levels (all *p* < 0.05) (Table 4).

Multivariable logistic regression analysis identified operative duration, POD1 AST, and POD1 lactate-to-albumin ratio as factors independently associated with the composite outcome (Table 5). In a sensitivity analysis restricted to patients undergoing laparoscopic sleeve gastrectomy (*n* = 257; 50 composite events), the principal associations remained materially unchanged: operative duration (adjusted OR 1.16 per 10 min, 95% CI 1.03–1.32; *p* = 0.016), POD1 LAR (adjusted OR 1.21 per 0.01-unit increase, 95% CI 1.03–1.41; *p* = 0.020), and POD1 AST (adjusted OR 1.14 per 10 U/L, 95% CI 1.06–1.23; *p* < 0.001) remained independently associated with the composite outcome, whereas BMI remained non-significant (adjusted OR 1.05, 95% CI 0.99–1.12; *p* = 0.107) (Supplementary Table S2).

**Figure 1:**
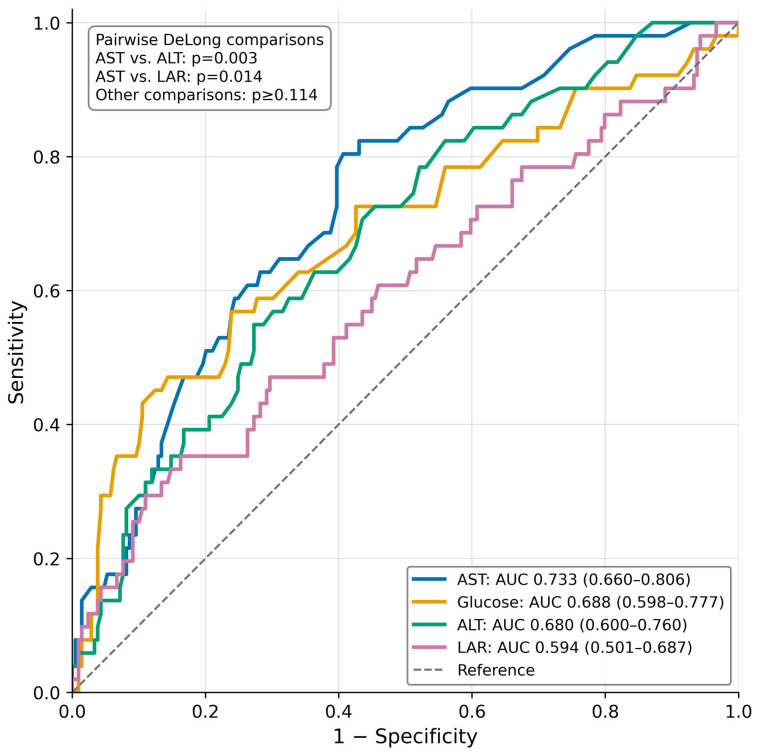
Receiver operating characteristic curves of POD1 biochemical markers for discrimination of the composite adverse perioperative outcome. AUC values are presented with DeLong 95% confidence intervals. Pairwise DeLong comparisons demonstrated significantly greater discrimination for AST than for ALT (*p* = 0.003) and LAR (*p* = 0.014); the remaining pairwise differences were not statistically significant.

The discriminative performance of the individual biochemical markers is presented in Table 6, and the corresponding ROC curves are shown in Figure 1. POD1 AST demonstrated the highest AUC (0.733, 95% CI 0.660–0.806), followed by POD1 glucose (AUC 0.688, 95% CI 0.598–0.777) and POD1 ALT (AUC 0.680, 95% CI 0.600–0.760), whereas POD1 LAR showed limited discrimination (AUC 0.594, 95% CI 0.501–0.687). Pairwise DeLong comparisons demonstrated that the AUC of AST was significantly higher than those of ALT (*p* = 0.003) and LAR (*p* = 0.014), but not significantly different from that of glucose (*p* = 0.320). None of the remaining pairwise comparisons reached statistical significance (all *p* ≥ 0.114).

## 4. Discussion

Obesity represents one of the most pressing healthcare challenges worldwide, and bariatric surgery remains the most effective intervention for achieving durable weight reduction and improvement in obesity-related comorbidities [12,13]. However, the growing volume of bariatric procedures has also increased the importance of perioperative risk stratification, as early postoperative complications continue to contribute to morbidity and healthcare resource utilization. The availability of simple and reliable predictors capable of identifying patients at increased risk of adverse outcomes could facilitate individualized perioperative care and further improve the safety profile of bariatric surgery. In this context, the prognostic value of readily available biochemical markers has attracted increasing attention.

In this retrospective cohort of 260 patients undergoing predominantly laparoscopic sleeve gastrectomy, a predefined composite adverse outcome occurred in 19.6% of cases and was driven mainly by postoperative intensive care unit admission. The early postoperative period was characterized by a uniform and statistically significant shift in inflammatory, hematological and metabolic parameters. Only a subset of these perturbations, however, carried independent prognostic weight: after multivariable adjustment, prolonged operative time, first postoperative day (POD1) aspartate aminotransferase (AST), and POD1 lactate-to-albumin ratio (LAR) were independently associated with the composite outcome. On receiver operating characteristic (ROC) analysis, POD1 AST showed the best discriminative ability (AUC 0.733), followed by POD1 glucose and ALT, whereas LAR—despite its independent association—demonstrated only limited stand-alone discrimination (AUC 0.594). Taken together, these findings suggest that early postoperative risk stratification may depend less on the magnitude of the global biochemical stress response and more on the specific pattern of organ-derived and perfusion-related signals, consistent with the growing emphasis on biomarker-guided perioperative risk assessment [5,8].

The pronounced POD1 changes observed in this study—rising white blood cell and neutrophil counts, CRP, NLR, lactate, transaminases and glucose, with concomitant falls in hemoglobin, platelet count, lymphocytes and albumin—are consistent with the expected neuroendocrine and inflammatory response to surgical trauma. Because this response was present in patients with and without complications, it underscores a central challenge in perioperative risk stratification: distinguishing the physiological background of uncomplicated recovery from the early biochemical signals associated with evolving complications [14]. Our findings suggest that markers reflecting tissue injury and perfusion, such as AST, LAR and glucose, may provide more clinically meaningful prognostic information than global inflammatory indices, which tend to increase predictably after surgery irrespective of outcome. This observation is consistent with recent evidence emphasizing the prognostic value of perioperative biomarker dynamics in bariatric surgery and the importance of distinguishing physiological postoperative responses from early signals of evolving complications [15,16].

POD1 AST emerged as the strongest individual predictor in our cohort. Early, transient transaminase elevation is a recognized phenomenon after laparoscopic abdominal surgery and is attributed chiefly to carbon dioxide pneumoperitoneum, which reduces hepatic and portal blood flow and produces relative hepatosplanchnic hypoperfusion, ischemia–reperfusion injury and oxidative stress [17]. Mechanical liver retraction and longer operative duration further amplify this effect; in laparoscopic gastric surgery, postoperative liver enzyme elevation has been linked to prolonged operative time and to the method and duration of liver retraction [18,19,20]. These mechanisms are particularly relevant to the bariatric population, since 62.3% of our patients had non-alcoholic fatty liver disease, and steatotic livers are known to be more susceptible to ischemia–reperfusion injury and oxidative stress [21]. A disproportionate POD1 AST rise may therefore reflect reduced hepatic reserve and greater intraoperative stress, potentially predisposing patients to a more complicated postoperative course. Although AST showed the highest AUC among the evaluated biomarkers, its discriminative performance remained moderate (AUC 0.733). The sensitivity of 80.4% at the internally derived 40 U/L cut-off suggests potential value for postoperative risk assessment; however, the corresponding specificity was only 59.3%. Therefore, AST should not be used as a stand-alone decision-making tool, and the proposed cut-off should be interpreted together with the patient’s perioperative clinical characteristics and other laboratory findings. AST elevation may reflect not only hepatic injury but also systemic tissue stress and ischemia–reperfusion injury. Supporting this broader interpretation, elevated postoperative AST has also been independently associated with early and late mortality after coronary artery bypass grafting, indicating that its prognostic relevance is not specific to bariatric surgery [22]. This systemic origin may partly explain its greater discriminative performance than ALT in our cohort.

LAR integrates two complementary dimensions of physiological stress: lactate, a marker of tissue hypoperfusion and anaerobic metabolism, and albumin, an indicator of nutritional reserve, systemic inflammation and hepatic synthetic capacity. Across critically ill and surgical populations—including sepsis, gastrointestinal bleeding and surgical or trauma intensive care cohorts—elevated LAR has been consistently associated with adverse outcomes [23,24]. Our results are concordant with previous reports, in that POD1 LAR remained independently associated with the composite outcome after adjustment (adjusted OR 1.20 per 0.01 increase). An important nuance, however, was that this independent association did not translate into strong stand-alone discrimination (AUC 0.594). Whereas multivariable models assess the incremental prognostic contribution of a marker after accounting for covariates, the AUC reflects its ability to discriminate outcomes across the entire population. In this relatively young, elective and low-acuity bariatric cohort, perioperative lactate values exhibited a narrow dynamic range and overt hypoperfusion was uncommon. At the internally derived cut-off of 0.0548, POD1 LAR demonstrated relatively high specificity (83.7%) but low sensitivity (35.3%). In clinical practice, an elevated LAR may therefore be interpreted as an adjunctive warning signal supporting reassessment of the patient’s hemodynamic and respiratory status, operative course, and other laboratory findings. Conversely, a value below this threshold cannot exclude an adverse perioperative course because most affected patients would not be identified by LAR alone. Accordingly, LAR should neither be used as a routine screening test nor serve as the sole basis for escalation-of-care decisions. Its role is better considered supportive and complementary to clinical assessment and more discriminative variables such as AST and operative duration. This interpretation differs from the stronger prognostic performance reported in critically ill populations and suggests that the clinical utility of LAR depends on patient acuity and clinical setting [23,24].

POD1 glucose levels were significantly higher in patients with adverse outcomes and showed moderate discriminatory ability (AUC 0.688). Consistent with previous reports, postoperative hyperglycemia may represent an integrated marker of surgical stress and has been associated with increased postoperative morbidity [25].

Prolonged operative time was an independent predictor of adverse outcomes (adjusted OR 1.17 per 10 min). This finding is consistent with registry-based studies demonstrating that longer operative duration is associated with increased rates of complications, readmission and reoperation following bariatric procedures [26]. Longer operations may reflect greater technical complexity, more extensive tissue manipulation and prolonged exposure to pneumoperitoneum and anesthesia, all of which may amplify hepatosplanchnic and inflammatory stress and potentially contribute to the parallel increase in AST observed in our cohort [18]. Importantly, operative time is an objective, routinely recorded intraoperative variable, and its prognostic value reinforces the concept that early risk is shaped by the surgical insult itself and not by baseline anthropometry alone. The procedure distribution should be considered when interpreting these findings. Although three patients underwent a gastric bypass procedure, the cohort almost exclusively represented laparoscopic sleeve gastrectomy. A sensitivity analysis restricted to sleeve gastrectomy produced materially unchanged estimates, indicating that the three bypass procedures did not drive the principal associations observed within this cohort. Although a large randomized trial reported similarly low overall perioperative complication rates following primary laparoscopic sleeve gastrectomy and Roux-en-Y gastric bypass, operative duration differed substantially between the procedures, supporting cautious procedure-specific interpretation [27]. Nevertheless, this analysis does not establish applicability to gastric bypass, revisional surgery, or open procedures. Gastric bypass involves intestinal reconstruction and anastomotic risks that differ from those of sleeve gastrectomy, while revisional procedures are generally characterized by adhesiolysis, greater technical complexity, and longer operative duration. Open surgery also eliminates carbon dioxide pneumoperitoneum, one of the proposed contributors to transient postoperative transaminase elevation. These procedural differences may alter both the magnitude and clinical meaning of POD1 AST and LAR changes. Consequently, the present estimates and cut-off values should be regarded as primarily applicable to laparoscopic sleeve gastrectomy until validated in adequately represented procedure-specific cohorts.

Although POD1 NLR increased markedly, it was not independently associated with adverse outcomes. This finding suggests that postoperative inflammatory indices may primarily reflect the universal response to surgical trauma rather than complication-specific processes. Therefore, organ- and perfusion-related biomarkers such as AST and LAR may provide more clinically relevant prognostic information in the early postoperative period. These findings do not necessarily contradict previous reports linking NLR to postoperative outcomes after bariatric surgery, but rather suggest that the prognostic value of NLR may depend on the timing of measurement and the clinical context, with postoperative measurements being more susceptible to the nonspecific effects of surgical stress [7,28].

The absence of an independent association between BMI and adverse outcomes supports the growing recognition that conventional anthropometric measures alone incompletely capture perioperative risk. Previous studies have demonstrated that more specific anatomical and physiological parameters may provide superior predictive information compared with BMI alone, while metabolic factors and perioperative biomarker dynamics have also been linked to postoperative outcomes after bariatric surgery [8,29,30].

From a practical perspective, AST, glucose, LAR, and operative duration may provide complementary information because they reflect different dimensions of perioperative stress, including tissue injury, metabolic response, perfusion–physiological reserve, and procedural burden. Integrating these variables into a combined clinical–biochemical model could potentially improve discrimination compared with the use of any single biomarker. However, the present study evaluated the discriminatory performance of individual biomarkers and used a parsimonious multivariable explanatory model; it was not designed to develop or validate a multivariable prediction score. Given the limited number of composite events, developing and evaluating a more complex combined model in the same cohort would carry a substantial risk of overfitting and optimistically biased performance estimates. Larger prospective studies should therefore develop multivariable models in dedicated derivation cohorts and assess their incremental value over AST alone in independent external validation cohorts.

First, the retrospective, single-center design and reliance on routinely collected data introduce the potential for selection bias, missing information, and residual confounding. In particular, detailed anesthetic exposure, the duration and magnitude of intraoperative hypotension, cumulative vasopressor dosage, and net fluid balance were not consistently available and could not be incorporated into the analyses. These factors may have influenced the postoperative biochemical measurements: anesthetic and vasoactive agents may affect systemic and hepatosplanchnic perfusion, hypotension may contribute to lactate and transaminase elevation, and fluid administration may alter albumin concentration through hemodilution and consequently affect LAR. Therefore, the observed associations cannot establish that the biomarker alterations were caused by the adverse perioperative course itself rather than by differences in intraoperative exposure and management. Second, despite the materially unchanged sleeve-only sensitivity analysis, the near-exclusive representation of laparoscopic sleeve gastrectomy limits procedure-level generalizability. Third, the number of composite events (*n* = 51) constrained the number of covariates that could be modeled reliably and reduced the precision of the ROC estimates. Fourth, biomarkers were assessed only at a single POD1 time point. Serial measurements and temporal trends may provide greater prognostic information than isolated values and therefore deserve further investigation. Moreover, postoperative ICU admission occurred in 41 of the 51 patients with a composite adverse outcome (80.4%). Consequently, the observed associations and ROC estimates were driven predominantly by ICU admission and should not be interpreted as demonstrating prediction of individual severe surgical complications such as anastomotic leak or infection. The small numbers of individual non-ICU events also precluded reliable component-specific multivariable analyses. Postoperative ICU admission was based on individualized clinical judgment and could be recommended independently by either the attending anesthesiologist or surgeon. The retrospective records did not allow reliable differentiation between ICU admissions anticipated preoperatively and unplanned admissions resulting from intraoperative or immediate postoperative events. Moreover, all ICU transfers occurred before POD1 blood sampling. Therefore, the associations between POD1 biomarkers and the composite outcome should not be interpreted as prospective prediction of ICU admission, but rather as associations with an adverse perioperative course predominantly characterized by the need for ICU care. Institution-specific admission thresholds and ICU availability may have influenced both the incidence and composition of the composite endpoint, limiting external generalizability. Finally, the ROC cut-offs were selected using the Youden index and evaluated in the same single-center cohort from which they were derived. Their sensitivity and specificity may therefore be optimistically estimated and may not be reproducible in populations with different patient characteristics, operative procedures, or ICU admission practices. Accordingly, the reported AST, glucose, ALT, and LAR thresholds should be considered exploratory and should not be used as clinical decision thresholds until they have undergone prospective external validation. Future multicenter studies should evaluate these cut-offs in independent cohorts with predefined outcomes and standardized sampling time points.

## 5. Conclusions

Among patients undergoing predominantly laparoscopic sleeve gastrectomy, longer operative duration and higher POD1 AST and LAR levels were independently associated with a composite adverse perioperative course dominated by immediate postoperative ICU admission. AST demonstrated the highest, although still moderate, individual discriminative performance, whereas LAR showed limited stand-alone discrimination and should be interpreted only as an adjunct to clinical and laboratory assessment. These findings are associative and do not establish prospective prediction of ICU admission or individual major surgical complications. Moreover, the internally derived cut-offs should not be applied as clinical decision thresholds. Prospective multicenter studies incorporating serial biomarker measurements, adequately represented procedure-specific cohorts, predefined clinically relevant outcomes, and independent external validation cohorts are required before these findings can be translated into routine clinical practice.

## Figures and Tables

**Table 1:** Baseline demographic and clinical characteristics of the study population.

Variable	Value (Mean ± SD or *n* (%))
Age, years	34.2 ± 10.1
BMI, kg/m^2^	46.0 ± 5.0
Male sex	42 (16.2%)
Current smoking	58 (22.3%)
Diabetes mellitus	22 (8.5%)
Hypertension	21 (8.1%)
Obstructive sleep apnea	17 (6.5%)
Coronary artery disease	6 (2.3%)
COPD/asthma	29 (11.2%)
NAFLD	162 (62.3%)
Hypothyroidism	11 (4.2%)
ASA I	4 (1.5%)
ASA II	91 (35.0%)
ASA III	165 (63.5%)
Operation time, min	62.2 ± 25.2
Sleeve gastrectomy	257 (98.8%)
Roux-en-Y gastric bypass	2 (0.8%)
One-anastomosis gastric bypass	1 (0.4%)
Laparoscopic approach	260 (100%)

Abbreviations: BMI, body mass index; COPD, chronic obstructive pulmonary disease; NAFLD, non-alcoholic fatty liver disease; ASA, American Society of Anesthesiologists physical status classification.

**Table 2:** Comparison of preoperative and POD1 laboratory parameters.

Parameter	Preoperative	POD1	*p* Value
Hemoglobin	13.31 ± 2.24	12.28 ± 1.64	<0.001
WBC	9.68 ± 4.26	13.63 ± 4.09	<0.001
Neutrophil	5.91 ± 1.86	11.28 ± 4.25	<0.001
Lymphocyte	2.72 ± 0.73	1.72 ± 1.05	<0.001
Platelet	343 ± 79	308 ± 80	<0.001
CRP	10.78 ± 8.70	30.22 ± 26.92	<0.001
Albumin	42.62 ± 2.78	38.10 ± 3.88	<0.001
Creatinine	0.66 ± 0.12	0.62 ± 0.14	<0.001
AST	24.27 ± 15.07	56.08 ± 66.93	<0.001
ALT	27.31 ± 20.09	61.51 ± 81.29	<0.001
Glucose	103.39 ± 28.32	118.54 ± 31.88	<0.001
Lactate	1.38 ± 0.49	1.72 ± 0.73	<0.001
NLR	2.31 ± 1.03	9.05 ± 6.35	<0.001
LAR	0.032 ± 0.012	0.046 ± 0.020	<0.001

Abbreviations: WBC, white blood cell count; CRP, C-reactive protein; AST, aspartate aminotransferase; ALT, alanine aminotransferase; NLR, neutrophil-to-lymphocyte ratio; LAR, lactate-to-albumin ratio; POD1, postoperative day 1.

**Table 3:** Early postoperative outcomes.

Outcome	*n* (%)
Postoperative ICU admission	41 (15.8)
Anastomotic leak	5 (1.9)
Surgical site/systemic infection	6 (2.3)
Length of hospital stay (days)	4.65 ± 2.09
30-day readmission	9 (3.5)
Composite adverse outcome	51 (19.6)

Abbreviations: ICU, intensive care unit. Data are presented as mean ± standard deviation or *n* (%). Composite adverse outcome was defined as the occurrence of at least one of the following events: postoperative ICU admission, anastomotic leak, surgical site/systemic infection, or 30-day readmission.

**Table 4:** Comparison between patients with and without composite adverse outcomes.

Variable	Composite Negative	Composite Positive	*p*
Operation time, min	60 (45–70)	65 (55–82.5)	0.015
WBC POD1	12.9 (10.8–15.0)	15.2 (11.7–18.8)	0.003
Creatinine POD1	0.60 (0.52–0.65)	0.65 (0.57–0.72)	0.005
AST POD1	33 (23–54)	64 (41–98.5)	<0.001
ALT POD1	38 (24–64)	61 (37.5–111)	<0.001
Glucose POD1	111 (98–125)	128 (109–160)	<0.001
NLR POD1	7.58 (4.10–12.69)	6.19 (3.99–13.58)	0.591
Lactate POD1	1.50 (1.20–2.00)	1.70 (1.30–2.15)	0.043
LAR POD1	0.0399 (0.033–0.052)	0.0442 (0.035–0.059)	0.038
LOS, days	4 (3–5)	5 (4–6)	<0.001

Abbreviations: WBC, white blood cell count; POD1, postoperative day 1; AST, aspartate aminotransferase; ALT, alanine aminotransferase; NLR, neutrophil-to-lymphocyte ratio; LAR, lactate-to-albumin ratio; LOS, length of hospital stay. Data are presented as median (interquartile range). *p* values were calculated using the Mann–Whitney U test.

**Table 5:** Multivariable logistic regression analysis.

Variable	Adjusted OR (95% CI)	*p* Value
BMI, per kg/m^2^	1.04 (0.98–1.11)	0.157
Operation time (per 10 min)	1.17 (1.04–1.31)	0.009
LAR POD1 (×100)	1.20 (1.04–1.38)	0.014
AST POD1 (per 10 U/L)	1.15 (1.07–1.24)	<0.001

Abbreviations: OR, odds ratio; CI, confidence interval; BMI, body mass index; LAR, lactate-to-albumin ratio; AST, aspartate aminotransferase; POD1, postoperative day 1.

**Table 6:** ROC analysis of POD1 biochemical markers for discrimination of the composite adverse perioperative outcome.

Marker	AUC (95% CI)	Best Cut-Off	Sensitivity (%)	Specificity (%)
AST POD1	0.733 (0.660–0.806)	40	80.4	59.3
ALT POD1	0.680 (0.600–0.760)	59	54.9	72.7
Glucose POD1	0.688 (0.598–0.777)	127	56.9	76.1
LAR POD1	0.594 (0.501–0.687)	0.0548	35.3	83.7

Abbreviations: POD1, postoperative day 1; ROC, receiver operating characteristic; AUC, area under the curve; LAR, lactate-to-albumin ratio; AST, aspartate aminotransferase; ALT, alanine aminotransferase.

## Data Availability

The datasets generated and analyzed during the current study are not publicly available due to patient privacy considerations and institutional regulations regarding the sharing of clinical data. However, anonymized data supporting the findings of this study are available from the corresponding author upon reasonable request and subject to approval by the relevant institutional authorities.

## Supplementary Materials

The following supporting information can be downloaded at https://www.mdpi.com/article/10.3390/diagnostics16152332/s1. Table S1. Univariable logistic regression analysis of factors associated with the composite adverse perioperative outcome. Table S2. Sensitivity analysis restricted to patients undergoing laparoscopic sleeve gastrectomy (*n* = 257).

## Abbreviations

ALT	alanine aminotransferase
ASA	American Society of Anesthesiologists physical status classification
AST	aspartate aminotransferase
AUC	area under the curve
BMI	body mass index
CI	confidence interval
COPD	chronic obstructive pulmonary disease
CRP	C-reactive protein
ICU	intensive care unit
LAR	lactate-to-albumin ratio
LOS	length of stay
NAFLD	non-alcoholic fatty liver disease
NLR	neutrophil-to-lymphocyte ratio
OR	odds ratio
POD1	postoperative day 1
ROC	receiver operating characteristic
WBC	white blood cell count

## References

[1] Noubiap J.J., Nansseu J.R., Nyaga U.F., Ndoadoumgue A.L., Ngouo A.T., Tounouga D.N., Tianyi F.-L., Foka A.J., Lontchi-Yimagou E., Nkeck J.R. (2025). Worldwide trends in metabolic syndrome from 2000 to 2023: A systematic review and modelling analysis. Nat. Commun..

[2] Xu Y., Jia W., Niu X., Wen J., Ji D., Li X., Liu M., Jiang S. (2025). Prevalence and Comorbidity Network Analysis of Obesity and Related Complications: A Real-World Study Based on 233,004 Individuals. Diabetes Metab. Syndr. Obes..

[3] Urones P., Juiz-Valiña P., Outeiriño-Blanco E., García-Brao M.J., Balboa-Barreiro V., Cordido F., Sangiao-Alvarellos S. (2025). Relevance of GDF15 as a biomarker for clinical outcomes after bariatric surgery. J. Endocrinol..

[4] Shoham G., Naveh S., Zoabi T., Gosher N., Messer N., Yuval J.B., Shnell M., Abu-Abeid A. (2026). Predictors of Postoperative Complications in Metabolic and Bariatric Surgery: A Retrospective Analysis Using Multivariable Logistic Regression. Medicina.

[5] Nenadic I., Stevanovic P., Bobos M., Stojanovic M., Dimic N., Bojic S., Dekic D., Radovanovic J., Djuric M. (2026). The Role of Biomarkers in Personalized Anesthesia: From Physiological Parameters to Molecular Diagnostics. Biomedicines.

[6] Da Silva M., Cleghorn M.C., Elnahas A., Jackson T.D., Okrainec A., Quereshy F.A. (2017). Postoperative day one neutrophil-to-lymphocyte ratio as a predictor of 30-day outcomes in bariatric surgery patients. Surg. Endosc..

[7] Alsabani M.H., Alenezi F.K., Alotaibi B.A., Alotaibi A.A., Olayan L.H., Aljurais S.F., Alarfaj N., Alkhurbush D., Almuhaisen G., Alkhmies L. (2024). Ratios of Neutrophils and Platelets to Lymphocytes as Predictors of Postoperative Intensive Care Unit Admission and Length of Stay in Bariatric Surgery Patients: A Retrospective Study. Medicina.

[8] AziziKia H., Shojaei S., Mousavi A., Salabat D., Shaker F., Dolama R.H., Radkhah H., Alilou S. (2024). Periprocedural Changes of Serum Biomarkers in Predicting Complications Following Bariatric Surgery for Obesity: Systematic Review and Meta-analysis. Obes. Surg..

[9] Li R., Zhu Y. (2025). Preoperative Hypoalbuminemia is Associated With Higher 30-day Mortality and Complications After Esophagectomy. Am. Surg..

[10] Lau K.K., Hsiao C.T., Fann W.C., Chang C.P. (2021). Utility of the Lactate/Albumin Ratio as a Predictor for Mortality in Necrotizing Fasciitis Patients. Emerg. Med. Int..

[11] Kasalović M., Odalović B., Mihajlović L., Jakovljević S., Elek Z., Igrutinović G., Anđelković M., Pajčin M. (2024). Prospective evaluation of serum and peritoneal fluid markers as indicators of postoperative complications in patients with enteric anastomosis. Ann. Saudi Med..

[12] Eisenberg D., Shikora S.A., Aarts E., Aminian A., Angrisani L., Cohen R.V., De Luca M., Faria S.L., Goodpaster K.P., Haddad A. (2022). 2022 American Society for Metabolic and Bariatric Surgery (ASMBS) and International Federation for the Surgery of Obesity and Metabolic Disorders (IFSO): Indications for Metabolic and Bariatric Surgery. Surg. Obes. Relat. Dis..

[13] De Luca M., Shikora S., Eisenberg D., Angrisani L., Parmar C., Alqahtani A., Aminian A., Aarts E., Brown W., Cohen R.V. (2024). Scientific Evidence for the Updated Guidelines on Indications for Metabolic and Bariatric Surgery (IFSO/ASMBS). Obes. Surg..

[14] Bedford J.P., Redfern O.C., O’Brien B., Watkinson P.J. (2025). Perioperative risk scores: Prediction, pitfalls, and progress. Curr. Opin. Anaesthesiol..

[15] Purdy J.C., Shatzel J.J. (2021). The hematologic consequences of obesity. Eur. J. Haematol..

[16] Spradley F.T. (2017). Metabolic abnormalities and obesity’s impact on the risk for developing preeclampsia. Am. J. Physiol. Regul. Integr. Comp. Physiol..

[17] Kuebler J.F., Schukfeh N., Vieten G., Osthaus W.A., Huber D., Dennhard N., Suempelmann R., Ure B.M., Metzelder M.L. (2018). Arterioportal shunting, splanchnic capillary perfusion, and the effects of colloids during capnoperitoneum in neonatal and adolescent pigs. Surg. Endosc..

[18] Sano A., Saito K., Kuriyama K., Nakazawa N., Ubukata Y., Hara K., Sakai M., Ogata K., Fukasawa T., Sohda M. (2021). Risk Factors for Postoperative Liver Enzyme Elevation After Laparoscopic Gastrectomy for Gastric Cancer. In Vivo.

[19] Goel R., Shabbir A., Tai C.M., Eng A., Lin H.Y., Lee S.L., Huang C.-K. (2013). Randomized controlled trial comparing three methods of liver retraction in laparoscopic Roux-en-Y gastric bypass. Surg. Endosc..

[20] Hiramatsu K., Aoba T., Kamiya T., Mohri K., Kato T. (2020). Novel use of the Nathanson liver retractor to prevent postoperative transient liver dysfunction during laparoscopic gastrectomy. Asian J. Endosc. Surg..

[21] Riazi K., Azhari H., Charette J.H., Underwood F.E., King J.A., Afshar E.E., Swain M.G., E Congly S., Kaplan G.G., Shaheen A.-A. (2022). The prevalence and incidence of NAFLD worldwide: A systematic review and meta-analysis. Lancet Gastroenterol. Hepatol..

[22] van Boxtel A.G.M., Bramer S., Soliman Hamad M.A., van Straten A.H.M. (2012). Perioperative serum aspartate aminotransferase level as a predictor of survival after coronary artery bypass grafting. Ann. Thorac. Surg..

[23] Song Z., Miao H., Xin X., Guo D., Deng Y., Wang H., Wang F. (2025). Elevated lactate-to-albumin ratio predicts short- and long-term mortality in trauma and surgical intensive care patients: A retrospective MIMIC-IV cohort study. Sci. Rep..

[24] Yang X., Ying S., Yuan X., Ying J., Yang J., Lv L. (2025). Lactate-to-albumin ratio as an independent predictor of mortality in critically ill patients with gastrointestinal bleeding: A retrospective cohort study. BMC Gastroenterol..

[25] Xiang Y., Luo T., Zeng L. (2025). Risk factors and clinical outcome of postoperative hyperglycemia after cardiac surgery with cardiopulmonary bypass. Front. Cardiovasc. Med..

[26] Clapp B., Marrero K., Corbett J., Sharma I., Hage K., Vierkant R.A., McKenzie T., Davis S.S., Ghanem O.M. (2023). Effect of operative times in bariatric surgery on outcomes: A matched analysis of the MBSAQIP database. Surg. Endosc..

[27] Hedberg S., Thorell A., Österberg J., Peltonen M., Andersson E., Näslund E., Hertel J.K., Svanevik M., Stenberg E., Neovius M. (2024). Comparison of Sleeve Gastrectomy vs Roux-en-Y Gastric Bypass: A Randomized Clinical Trial. JAMA Netw. Open.

[28] Liu C., Zheng J., Bai Y., Gan L., Gu Y. (2025). Neutrophil-to-lymphocyte ratio for predicting postoperative mortality after hip fracture surgery: A systematic review and meta-analysis. J. Orthop. Surg. Res..

[29] Sarna M.J., Giorgi M., Luhrs A.R. (2022). Metabolic syndrome as a predictor of perioperative outcomes in primary bariatric surgery, a MBSAQIP survey. Surg. Endosc..

[30] Altınsoy K.E., Bayhan B.U. (2025). Ultrasound-Measured Skin-to-Epiglottis Distance as a Predictor of Difficult Intubation in Obese Patients: A Prospective Observational Study. J. Clin. Med..

