# Supplementary material for: Association of Early Postoperative Biochemical Markers with a Composite Adverse Perioperative Outcome After Bariatric Surgery
Source: Diagnostics (Basel). 2026 Jul 25;16(15):2332. doi: 10.3390/diagnostics16152332 (PMC13464782; doi:10.3390/diagnostics16152332)
Supplement: Supplementary file 1 [file diagnostics-16-02332-s001.zip › Supplementary Table S1.pdf]

Supplementary Table S1. Univariable logistic regression analysis of factors associated with the composite adverse perioperative outcome.

Variable	n	Unadjusted OR (95% CI)	p value
**Age, per year**	260	1.03 (1.00–1.06)	0.099
**Male sex (vs. female)**	260	2.12 (1.01–4.46)	**0.047**
**BMI, per kg/m²**	260	1.05 (0.99–1.12)	0.080
**ASA physical status, per class**	260	1.14 (0.62–2.09)	0.668
**Diabetes mellitus (yes vs. no)**	260	0.90 (0.29–2.79)	0.860
**Hypertension (yes vs. no)**	260	2.81 (1.10–7.18)	**0.032**
**Obstructive sleep apnea (yes vs. no)**	260	1.28 (0.40–4.11)	0.675
**NAFLD (yes vs. no)**	260	2.00 (1.01–3.99)	**0.048**
**Operative duration, per 10 min**	260	1.17 (1.04–1.31)	**0.007**
**Hemoglobin POD1, per 1 g/dL**	260	1.27 (1.05–1.54)	**0.014**
**WBC POD1, per 1 × 10^3/μL**	260	1.14 (1.06–1.23)	**<0.001**
**CRP POD1, per 10 mg/L**	260	0.96 (0.85–1.09)	0.540
**Albumin POD1, per 1 g/L**	260	0.98 (0.90–1.06)	0.579
**Creatinine POD1, per 0.1 mg/dL**	260	1.40 (1.12–1.74)	**0.003**
**Lactate POD1, per 1 mmol/L**	260	1.76 (1.19–2.60)	**0.004**
**NLR POD1, per 1-unit increase**	260	1.00 (0.95–1.05)	0.934
**LAR POD1, per 0.01-unit increase**	260	1.22 (1.06–1.40)	**0.006**
**AST POD1, per 10 U/L**	260	1.16 (1.08–1.24)	**<0.001**
**ALT POD1, per 10 U/L**	260	1.10 (1.04–1.17)	**<0.001**
**Glucose POD1, per 10 mg/dL**	260	1.20 (1.09–1.32)	**<0.001**

Data are derived from separate univariable binary logistic regression models. The composite outcome was defined as the occurrence of at least one of the following: immediate postoperative ICU admission, anastomotic leak, postoperative infection, or 30-day hospital readmission. ORs for categorical variables compare the category shown with the stated reference category. p values <0.05 are shown in bold.

Abbreviations: ALT, alanine aminotransferase; ASA, American Society of Anesthesiologists; AST, aspartate aminotransferase; BMI, body mass index; CI, confidence interval; CRP, C-reactive protein; ICU, intensive care unit; LAR, lactate-to-albumin ratio; NAFLD, non-alcoholic fatty liver disease; NLR, neutrophil-to-lymphocyte ratio; OR, odds ratio; POD1, postoperative day 1; WBC, white blood cell count.
