# Supplementary material for: Association of Early Postoperative Biochemical Markers with a Composite Adverse Perioperative Outcome After Bariatric Surgery
Source: Diagnostics (Basel). 2026 Jul 25;16(15):2332. doi: 10.3390/diagnostics16152332 (PMC13464782; doi:10.3390/diagnostics16152332)
Supplement: Supplementary file 1 [file diagnostics-16-02332-s001.zip › Supplementary Table S2.pdf]

Supplementary Table S2. Sensitivity analysis restricted to patients undergoing laparoscopic sleeve gastrectomy (n=257).

Variable	Adjusted OR (95% CI)	p value
**BMI, per kg/m²**	1.05 (0.99–1.12)	0.107
**Operative duration, per 10 min**	1.16 (1.03–1.32)	**0.016**
**LAR POD1, per 0.01-unit increase**	1.21 (1.03–1.41)	**0.020**
**AST POD1, per 10 U/L**	1.14 (1.06–1.23)	**<0.001**

The sensitivity analysis included 257 patients and 50 composite outcome events. All four covariates were entered simultaneously into the multivariable logistic regression model.

Abbreviations: AST, aspartate aminotransferase; BMI, body mass index; CI, confidence interval; LAR, lactate-to-albumin ratio; OR, odds ratio; POD1, postoperative day 1.
